# Ion-Enhanced Etching Characteristics of sp^2^-Rich Hydrogenated Amorphous Carbons in CF_4_ Plasmas and O_2_ Plasmas

**DOI:** 10.3390/ma14112941

**Published:** 2021-05-29

**Authors:** Jie Li, Yongjae Kim, Seunghun Han, Heeyeop Chae

**Affiliations:** 1 School of Chemical Engineering, Sungkyunkwan University (SKKU), Suwon 16419, Korea; lj870408@skku.edu (J.L.); hsh12040@naver.com (S.H.); 2 Sungkyunkwan Advanced Institute of Nanotechnology (SAINT), Sungkyunkwan University (SKKU), Suwon 16419, Korea; yon521@skku.edu

**Keywords:** sp^2^-rich hydrogenated amorphous carbon, ion-enhanced etching, CF_4_ and O_2_ plasma etching

## Abstract

The sp^2^-rich hydrogenated amorphous carbon (a-C:H) is widely adopted as hard masks in semiconductor-device fabrication processes. The ion-enhanced etch characteristics of sp^2^-rich a-C:H films on ion density and ion energy were investigated in CF_4_ plasmas and O_2_ plasmas in this work. The etch rate of sp^2^-rich a-C:H films in O_2_ plasmas increased linearly with ion density when no bias power was applied, while the fluorocarbon deposition was observed in CF_4_ plasmas instead of etching without bias power. The etch rate was found to be dependent on the half-order curve of ion energy in both CF_4_ plasmas and O_2_ plasmas when bias power was applied. An ion-enhanced etching model was suggested to fit the etch rates of a-C:H in CF_4_ plasmas and O_2_ plasmas. Then, the etch yield and the threshold energy for etching were determined based on this model from experimental etch rates in CF_4_ plasma and O_2_ plasma. The etch yield of 3.45 was observed in CF_4_ plasmas, while 12.3 was obtained in O_2_ plasmas, owing to the high reactivity of O radicals with carbon atoms. The threshold energy of 12 eV for a-C:H etching was obtained in O_2_ plasmas, while the high threshold energy of 156 eV was observed in CF_4_ plasmas. This high threshold energy is attributed to the formation of a fluorocarbon layer that protects the a-C:H films from ion-enhanced etching.

## 1. Introduction

Recently, carbons at the nanoscale attract great interest for a huge number of applications, such as transistor, field emission display, actuator, molecular wires/interconnect, transparent conducting film, supercapacitor, and catalyst [1,2,3,4,5,6,7,8]. Especially hydrogenated amorphous carbon (a-C:H) layers are widely used as a hard mask in semiconductor-device fabrication processes. The a-C:H layers are replacing conventional organic photoresists in high-aspect-ratio etching processes because they can provide a higher etch resistance and etch selectivity than photoresists to various harsh etching chemicals, such as fluorocarbon plasmas [9,10,11]. Moreover, a-C:H films are also attractive because they are easy to be removed with oxygen plasmas when they are compared with other non-carbon inorganic mask materials. The a-C:H hard mask contains sp^2^ carbons and sp^3^ carbons, and its sp^2^/sp^3^ ratio is strongly related to the physical and chemical properties of a-C:H hard mask films [12,13,14,15,16,17]. In general, higher film density, stronger film hardness, and higher etching resistance are observed in higher sp^2^/sp^3^ ratio of a-C:H films. Therefore, a-C:H films with high sp^2^/sp^3^ ratio, or sp^2^-rich a-C:H films are preferred over sp^3^-rich a-C:H for hard mask applications in semiconductor processes [9,18].

The understanding of etching characteristics and mechanism of sp^2^-rich a-C:H hard mask in plasmas is required for highly selective high-aspect-ratio etching processes, and the etching characteristics have been studied by several research groups [19,20,21,22]. The etching of sp^2^-rich a-C:H can be characterized by the etch yield and the threshold energy. The etch yield is defined as the number of carbon atoms removed by each incident ion and is affected by the reactivity of ions, ion energy, and surface composition. The threshold energy is the minimum energy of ion required for carbon removal and is affected by the reactivity of etched films. A relatively low etch yield of 0.26 at ion energy of 400 eV was reported for sp^2^-rich a-C:H by physical sputtering in non-reactive Ar plasmas [19]. A lot higher etch yield of 4.3 was reported for sp^2^-rich a-C:H films when the carbon films were exposed to Ar^+^ ions and O_2_, indicating the O_2_ molecules enhance etch reaction of carbons significantly [20]. Hansen et al. determined the threshold energy of a-C:H for etching in a cascaded arc Ar/H_2_ plasma and they reported the significant decrease of threshold energy to 3–5 eV with H radicals from 58 eV without H radicals [21]. Salonen et al. reported a high etch yield of 1.0 and a low threshold energy of 1.0 eV in a mixture of H^+^ ions and H atoms, using classical molecular-dynamics simulations [22]. Fluorocarbon plasmas are typically adopted for patterning processes with a-C:H hard masks, and O_2_ plasmas are applied for the removal of residual a-C:H hard masks in many dielectric etch processes, but few research studies on a-C:H etching have been reported in theses plasmas [23,24]. In addition, the direct effect of ions on a-C:H etching in fluorocarbon and O_2_ plasmas is hardly studied.

In this work, the effect of ions on the sp^2^-rich a-C:H etch was investigated in CF_4_ plasmas and O_2_ plasmas by measuring etch rates at different ion density and ion energy. An ion-enhanced etching model was suggested to fit the etch mechanism of sp^2^-rich a-C:H films in CF_4_ plasmas and O_2_ plasmas. The etch yield and threshold energy were determined from experimental etch rates fitted on this model, and the etch yield and threshold energy were compared with those reported in previous researches in various conditions. The chemical compositions of a-C:H films after etching in CF_4_ and O_2_ plasmas were also investigated.

## 2. Experimental Setup and Procedures

**Figure 1:**
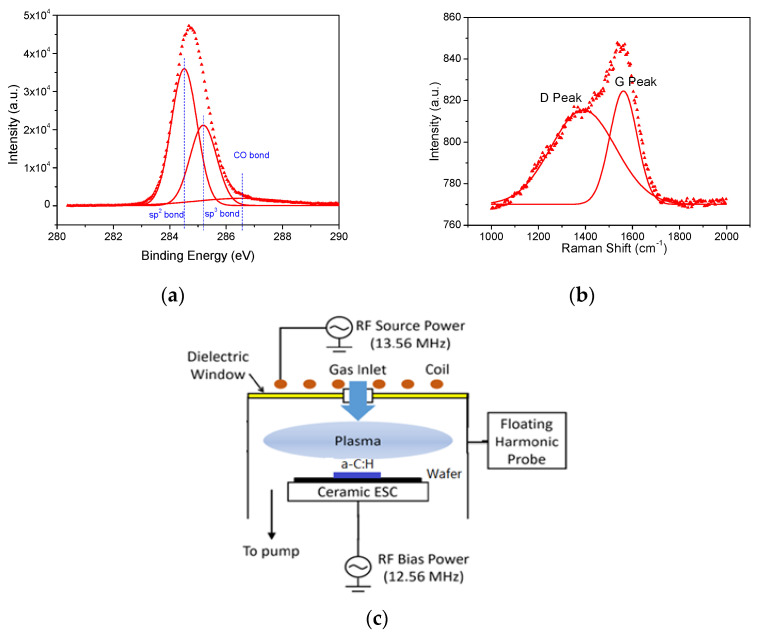
(**a**) XPS C1s profile of a-C:H films deposited in the C_2_H_2_ ICP plasma. C1s spectrum of a-C:H was deconvoluted into three bands (sp^2^ bands, sp^3^ bands, and CO bands) by Gaussian fitting. (**b**) Raman spectrum of a-C:H films. Raman spectrum of a-C:H was deconvoluted into D and G peaks by Gaussian fitting. (**c**) Schematic diagram of ICP chamber used in this work.

The a-C:H films used in this work were deposited in a C_2_H_2_ plasma in an inductively coupled plasma (ICP) reactor with 250 W power and 21 mTorr process pressure. The a-C:H films of about 600 nm were obtained after deposition of 15 min and were determined by ellipsometry. The a-C:H films were characterized by X-ray photoelectron spectroscopy (XPS), where the C 1s peak was deconvoluted into three peaks of the sp^2^ (284.5 eV), sp^3^ (285.2 eV), and CO band (286.5 eV) by Gaussian fitting, as shown in Figure 1a. The sp^2^/sp^3^ ratio of a-C:H films was 1.70 determined by the relative intensity of the sp^2^ versus sp^3^ peak, and is similar with the number reported in a previous research [9]. The a-C:H films were also characterized by Raman spectroscopy and the Raman spectrum was deconvoluted into D and G peaks by Gaussian fitting, as shown in Figure 1b. The intensity ratios of the D and G peaks (*I_D_/I_G_*) in the Raman spectrum can indicate the sp^2^/sp^3^ ratio, because the D peak contains information on sp^2^ carbons only, while the G peak provides information on both sp^2^ and sp^3^ carbons [25,26]. An *I_D_/I_G_* of 2.0 was obtained in a-C:H films; that is similar to the *I_D_/I_G_* reported in previous researches [26].

The sp^2^-rich a-C:H films were etched in CF_4_ and O_2_ plasmas in another ICP chamber, as shown in Figure 1c. This ICP reactor was operated with a source power of 13.56 MHz radio frequency (RF) and a bias power of 12.56 MHz. The source power was delivered through an inductive coil on the top of a quartz window and the bias power was delivered through a bottom electrode. The a-C:H films were placed on the center of a 100 mm silicon wafer and on the top of a ceramic electrostatic chuck, which was positioned on the bottom electrode. The reactor was evacuated to 5 mTorr before introducing the etching gas, and after introducing 20 sccm CF_4_ or O_2_, the reactor pressure was maintained at 100 mTorr. CF_4_ plasmas and O_2_ plasmas were generated with the source power of 50~250 W and bias power of 0~50 W, and sp^2^-rich a-C:H films were etched in these plasmas for 1 min. The chemical compositions of sp^2^-rich a-C:H films were investigated after 1 min CF_4_ and O_2_ plasma etching with 250 W source power and 10 W bias power.

The ion density in plasmas was measured by using a floating harmonic probe (WiseProbe, P&A solutions, Seoul, Korea), and the bias voltage was determined by a VI probe (Octiv Poly, Impedans, Dublin, Ireland). The etch rates of a-C:H films were determined by measuring the thickness of the a-C:H films with an ellipsometer (SE MF-1000, NanoView, Ansan, Korea) before and after etching processes. The chemical compositions of a-C:H films before and after etching processes were analyzed with XPS (Escalab 250, Thermo-Scientific, Waltham, MA, USA).

## 3. Results and Discussion

**Figure 2:**
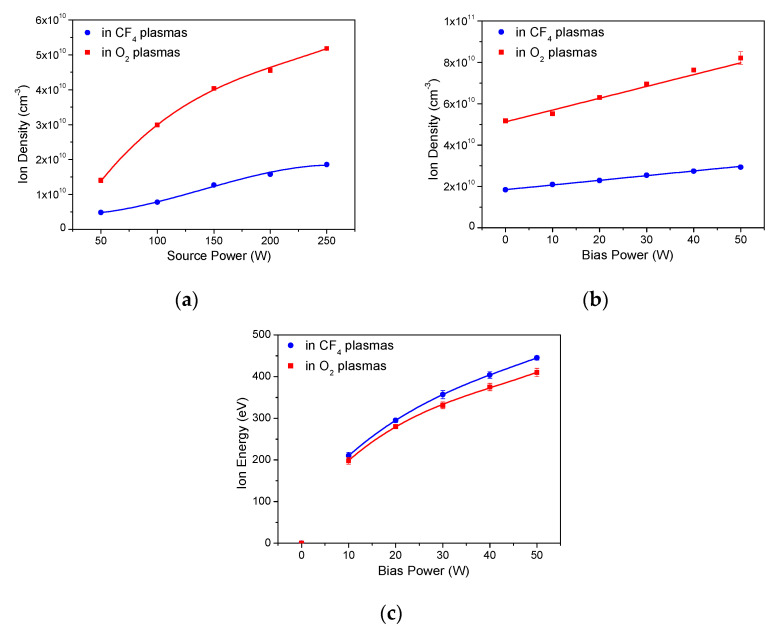
(**a**) Ion density in CF_4_ plasmas and O_2_ plasmas without bias power as a function of source power. (**b**) Ion density in CF_4_ plasmas and O_2_ plasmas with 250 W source power as a function of bias power. (**c**) Ion energy in CF_4_ plasmas and O_2_ plasmas with 250 W source power as a function of bias power.

Ion density and ion energy were determined for the plasmas generated with the varied source power and bias power, and these values are plotted in Figure 2 as a first step. The ion density is 2.5–3.0 times higher in O_2_ plasmas than in CF_4_ plasmas, as shown in Figure 2a,b, and this is attributed to the low ionization energy of oxygen atoms (13.6 eV) compared with that of fluorine atoms (17.4 eV). The ion density increases by 3.9 and 3.7 times in CF_4_ and O_2_ plasmas in the source power range of 50~250 W, respectively. The ion density increases about 60% in both CF_4_ and O_2_ plasmas with bias power in the range of 0~50 W, indicating that ion density is affected more significantly by the source power than bias power. The ion energy in plasmas was estimated from the bias voltage as shown in Figure 2c. The effect of source power on ion energy is marginal in ICP plasmas [27], and the ion energy increases by 2.1 times in both CF_4_ and O_2_ plasmas with increased bias power from 10 to 50 W. The ion energy in CF_4_ plasmas is slightly higher than that in O_2_ plasmas probably due to the higher sheath potential resulting from the lower ion density in CF_4_ plasmas.

**Figure 3:**
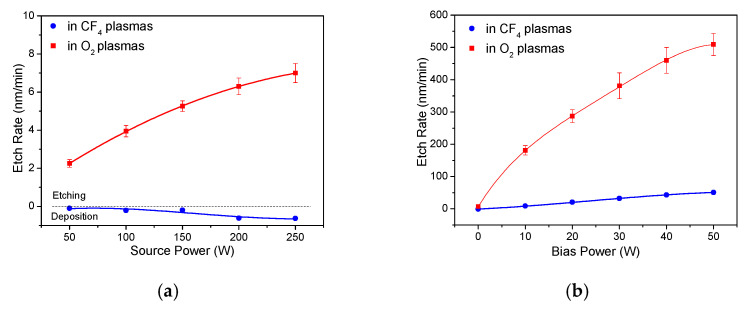
(**a**) Etch rates of sp^2^-rich a-C:H in plasmas without bias power as a function of source power. (**b**) Etch rates of sp^2^-rich a-C:H in plasmas with 250 W source power as a function of bias power.

The etch-rate dependences of sp^2^-rich a-C:H on source power and bias power were investigated in CF_4_ plasmas and O_2_ plasmas shown in Figure 3 as the next step. The etch rate of a-C:H in O_2_ plasmas increases from 2.3 to 7.0 nm/min with increased source power even without bias power as shown in Figure 3a. This is attributed to the increased density of O radicals in O_2_ plasmas with the increased source power. The O radicals are expected to be chemisorbed on the surface of a-C:H films with the low activation energy of 0.046 eV and react with carbons for carbon removal with the activation energy of 0.28 eV by oxidization in O_2_ plasmas [28,29]. In CF_4_ plasmas, slight film deposition was observed on the surface of a-C:H films instead of etching when no bias power was applied. The fluorocarbon deposition was also reported on the outer and inner surface of carbon nanotube in CF_4_ plasmas [30]. The F radicals are easily chemisorbed on carbon surface with a low activation energy of 0.13 eV, but it was reported that it is difficult to remove the carbons due to the high activation energy of 0.56–2.4 eV for removal reactions [31]. The etch rate of a-C:H was measured with bias power, as shown in Figure 3b. The etch rate increases from 0 to 51 nm/min when the bias power increases from 0 to 50 W in CF_4_ plasmas. Ions are accelerated and bombarded with the bias power, and the ions break C-C and C-H bonds and create dangling bonds on the surface of films, which allows the carbon removal with F radicals with low activation energy. A sharp increase of etch rate of sp^2^-rich a-C:H from 7.0 to 510 nm/min was observed with increased bias power in the range of 0~50 W in O_2_ plasmas, indicating the etching is accelerated with the energetic ions.

**Figure 4:**
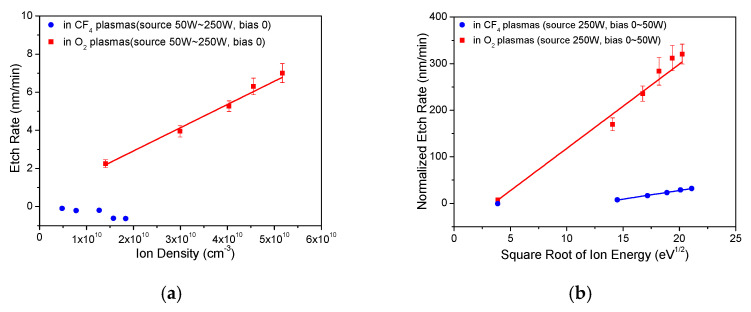
(**a**) Etch rates of sp^2^-rich a-C:H as a function of ion density. (**b**) Normalized etch rates of sp^2^-rich a-C:H as a function of square root of ion energy.

The etch rate of sp^2^-rich a-C:H was correlated with ion density and ion energy separately to investigate the etching mechanism in CF_4_ plasmas and O_2_ plasmas, as shown in Figure 4. The etch rate of sp^2^-rich a-C:H increases linearly with ion density in O_2_ plasmas, as shown in Figure 4a. The creation of dangling bonds by bombarding ions accelerates the carbon reaction with O radicals [23]. The etch rate is affected by ion energy and ion density obviously, and it was normalized to exclude the effect of ion density by dividing the etch rate with the ratio of ion density with and without bias power, as shown in Equation (1):

(1) $${\mathrm{ER}}_{\mathrm{normalized}}={\mathrm{ER}}_{\mathrm{orginal}}*\frac{n_{i}\left(\mathrm{without}\;\mathrm{bias}\;\mathrm{power}\right)}{n_{i}\left(\mathrm{with}\;\mathrm{bias}\;\mathrm{power}\right)}$$

where ER is the etch rate, and n_i_ is the ion density in plasmas. The effect of ion energy on the etch rate of sp^2^-rich a-C:H films is plotted in Figure 4b. The normalized etch rate of sp^2^-rich a-C:H fits well with half-order curves of ion energy in CF_4_ plasmas and O_2_ plasmas. The ions of high energy help in breaking C–C and C–H bonds and creating dangling bonds on the surface of films in each bombarding, and active radicals react with these dangling bonds, resulting in the etching of carbon atoms [32]. This significant enhancement of etch rate with bias power indicates that the creation of dangling bonds by energetic ions is believed to be limiting factor in the carbon removal.

**Figure 5:**
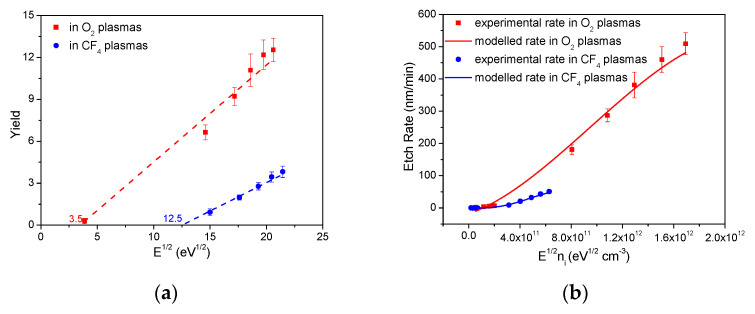
(**a**) Etch yield of sp^2^-rich a-C:H as a function of E^1/2^ in CF_4_ plasmas and O_2_ plasmas. E is the ion energy. (**b**) Etch rates of sp^2^-rich a-C:H as a function of E^1/2^n_i_ in CF_4_ plasmas and O_2_ plasmas. n_i_ is the ion density. Experimental data and modeling data are listed for comparison.

A model of ion-enhanced etching was developed with the following equations and plotted in Figure 5 [33].

(2) R=YI/ρ

where R is the etch rate, Y is the etch yield, I is the ion flux, and ρ is the density of a-C:H (1.9 g/cm^3^) [9]. The etch yield of sp^2^-rich a-C:H calculated by Equation (2) increases linearly with the square root of ion energy in CF_4_ plasmas and O_2_ plasmas, as shown in Figure 5a. The etch yield of 3.45 and 12.3 was obtained in CF_4_ and O_2_ plasmas, respectively, at 400 eV. The etch yield, Y, can be described by Equation (3) [34]:

(3) $$Y=b\left(E^{\frac{1}{2}}-E_{\mathrm{th}}^{\frac{1}{2}}\right)$$

where b is the proportional parameter, E is the ion energy, and E_th_ is the threshold energy. The coefficients b and E_th_ in Equation (3) were determined as the slope and the horizontal intercept in the etch yield and ion energy plot, as shown in Figure 5a and Table 1. The etch rates of sp^2^-rich a-C:H were modeled with these b and E_th_, and this model agrees well with experimental etch rates, as shown in Figure 5b, indicating that the ion-enhanced etching fits the etching mechanism of sp^2^-rich a-C:H in CF_4_ plasmas and O_2_ plasmas well.

**Figure 6:**
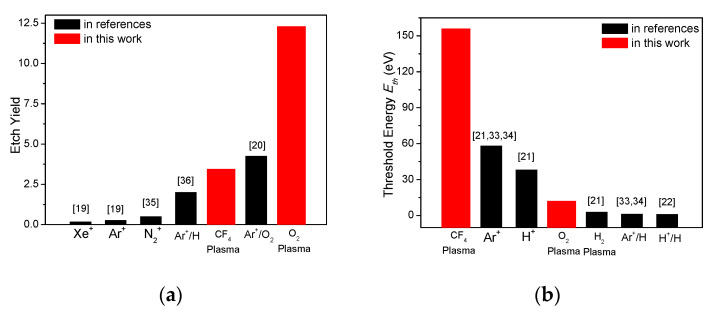
(**a**) The etch yield in CF_4_ plasmas and O_2_ plasmas in this work and other systems reported in references. The yield was collected with electron energy of 400 eV. (**b**) The threshold energy in CF_4_ plasmas and O_2_ plasmas in this work and other systems reported in references.

The etch yield and the threshold energy obtained in CF_4_ plasmas and O_2_ plasmas were compared with those reported values in previous works of carbon etching with Ar^+^ ions only, Ar^+^ ions/O_2_ molecules and Ar^+^ ions/H radicals [19,20,21,22,32,35,36,37,38]. The etch yield at 400 eV is plotted in Figure 6a from the results of these researches. The highest etch yield (12.3) was observed in O_2_ plasmas among these systems, due to the extremely high activity of O radicals with carbon atoms. The activation energy of carbon removal is as low as 0.28 eV in O_2_ plasmas, as mentioned earlier [29]. The etch yield in CF_4_ plasmas (3.45) is lower than that in Ar^+^ ions/O_2_ molecules (4.25), but higher than that in Ar^+^ ions/H radicals (2), indicating the reactivity of F radicals with carbons is lower than O_2_ molecules and higher than H radicals. This is also proven with the activation energy of carbon removal in CF_4_ plasma (0.56–2.4 eV), which is higher than that of carbon reaction with O_2_ molecules (0.46 eV) and lower than that of carbon removal with H radicals (1.6–2.5 eV) [21,38]. The relatively low etch yield was also found in carbon etching with inert Xe^+^, Ar^+^, and N_2_^+^ without any radical/reactive molecules on surface. The threshold energy measurements of a-C:H in CF_4_ plasmas and O_2_ plasmas are notated with those in different environments, in Figure 6b. The quite high threshold energy of 156 eV was observed in CF_4_ plasmas, and it is attributed to the formation of fluorocarbon layer on the surface of sp^2^-rich a-C:H films. This fluorocarbon layer is expected to protect a-C:H films from ion-enhanced etching by protecting C-C bonds. The threshold energies reported in H^+^ ions/H radicals (1 eV), Ar^+^ ions/H radicals (1.3 eV), and H_2_ plasmas (3 eV) are lower than that in O_2_ plasmas (12 eV) of this work. H radicals are smaller than O radicals, making them more easily penetrate into sp^2^-rich a-C:H films. These H radicals in a-C:H films are believed to transfer sp^2^-carbons into sp^3^-carbons by forming C-H bonds and decrease the bonding energy in a-C:H films, resulting in the decrease of etch threshold energy.

**Figure 7:**
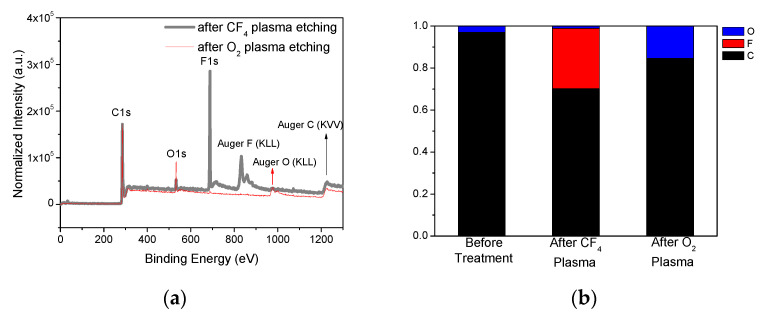

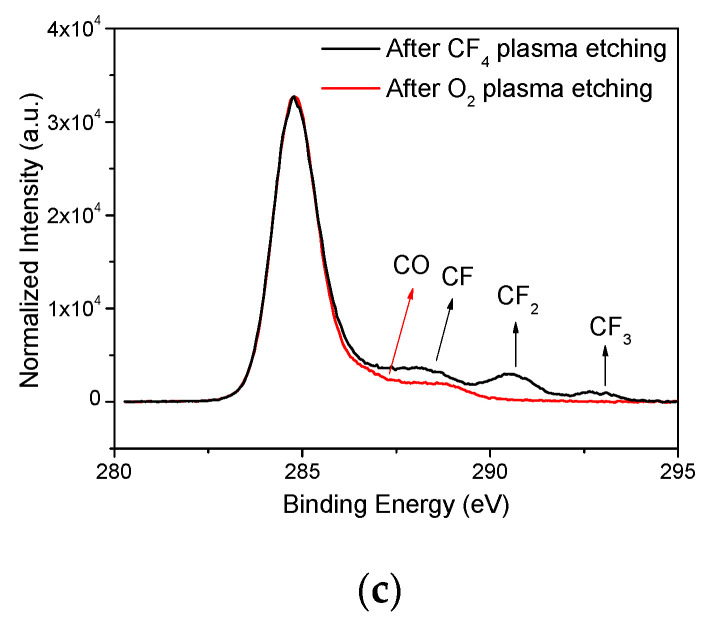
(**a**) XPS of a-C:H after CF_4_ and O_2_ plasmas etching, (**b**) chemical compositions of a-C:H before and after plasmas etching, and (**c**) XPS C1s profiles of a-C:H after CF_4_ and O_2_ plasmas etching.

Chemical compositions of a-C:H films were investigated after CF_4_ plasmas and O_2_ plasmas etching with XPS analysis, as shown in Figure 7a. The chemical compositions of a-C:H films were determined from the peak areas and are presented in Figure 7b. In total, 29% of fluorine was found to be included in a-C:H films after CF_4_ plasma etching; that is higher than oxygen atomic percentage of 15% in a-C:H films after O_2_ plasma etching. The fluorocarbon layers on the surface of carbon films are expected to form in CF_4_ plasmas, but residual oxygen atoms are more difficult to stay in carbon films after O_2_ plasma etching. Similar phenomena were reported in the carbon nanotube exposed to CF_4_ plasma and O_2_ plasma [30]. The CF, CF_2_, and CF_3_ bonds are observed in XPS C1s spectra after CF_4_ plasmas etching, as shown in Figure 7c, indicating the formation of fluorocarbon layer.

## 4. Conclusions

Etch characteristics of sp^2^-rich a-C:H films were investigated with different ion density and ion energy in CF_4_ plasmas and O_2_ plasmas. The etch rate of sp^2^-rich a-C:H films increases linearly with ion density in O_2_ plasmas, while no etch was observed in CF_4_ plasmas when no bias power was applied. The a-C:H etch rates in CF_4_ plasmas and O_2_ plasmas fit well with the half-order curve of ion energy with bias power applied. An ion-enhanced etching model was suggested to fit the etch rates, and the etch yield and threshold energy were estimated from this model. The etch yields of 3.45 in CF_4_ plasmas and 12.3 in O_2_ plasmas were obtained. The high etch yield in O_2_ plasmas is attributed to the high reactivity of O radicals with carbon atoms. The etch threshold energy values of 156 eV in CF_4_ plasmas and 12 eV in O_2_ plasmas were observed. The high threshold energy in CF_4_ plasmas is attributed to the formation of fluorocarbon layers on the surface of sp^2^-rich a-C:H films that protects the films from ion-enhanced etching.

## Figures and Tables

**Table 1:** Coefficients in ion-enhanced etch modeling used in this work.

a-C:H	b(eV^−1/2^)	$E_{th}(\mathrm{eV})$
in CF_4_ plasmas	0.4	156
in O_2_ plasmas	0.65	12

## Data Availability

The data presented in this study are available on request from the corresponding author. The data are not publicly available due to privacy.
